# Exploring the perspectives of people post-stroke, carers and healthcare professionals to inform the development of an intervention to improve cognitive impairment post-stroke

**DOI:** 10.12688/hrbopenres.13184.1

**Published:** 2020-12-22

**Authors:** Mairéad O' Donoghue, Pauline Boland, Siobhan Leahy, Rose Galvin, Sara Hayes

**Affiliations:** 1 School of Allied Health, University of Limerick, Limerick, V94 X5K6, Ireland; 2 Health Research Institute, University of Limerick, Limerick, V94 T9PX, Ireland; 3 Ageing Research Centre, University of Limerick, Limerick, V94 X5K6, Ireland

**Keywords:** Stroke, cognition, cognitive impairment, rehabilitation, qualitative, interviews

## Abstract

**Background**: Stroke is a leading cause of death and disability worldwide. Cognitive impairment is common post-stroke and can result in negative sequalae such as a lower quality of life, increased carer burden and increased healthcare costs. Despite the prevalence and associated burden of post-stroke cognitive impairment, there is uncertainty regarding the optimum intervention to improve cognitive function post-stroke. By exploring the perspectives of people post-stroke, carers and healthcare professionals on cognitive impairment, this qualitative study aims to inform the design and development of an intervention to rehabilitate cognitive impairment post-stroke.

**Methods**: A qualitative descriptive approach will be applied, using semi-structured interviews with people post-stroke, carers and healthcare professionals. People post-stroke will be recruited via gatekeepers from a local stroke support group and Headway, a brain injury support service. Carers will be recruited via a gatekeeper from a local carers branch. Healthcare professionals will be recruited via gatekeepers from relevant neurological sites and via Twitter. The final number of participants recruited will be guided by information power. Data will be collectively analysed and synthesised using thematic analysis. The Consolidated Criteria for Reporting Qualitative Studies (COREQ) guidelines will be used to standardize the conduct and reporting of the research.

**Conclusions**: It is anticipated that exploring the perspectives of people post-stroke, carers and healthcare professionals on cognitive impairment post-stroke will inform the development of an evidence-based optimal intervention to rehabilitate cognitive deficits post-stroke. This study was granted ethical approval from the Faculty of Education and Health Sciences Research Ethics Committee at the University of Limerick. Study findings will be disseminated locally through presentations at stroke support groups, as well as internationally through academic conferences and peer-reviewed journals.

## Introduction

Stroke is one of the leading causes of death and disability adjusted life years on a global scale ^1^. Cognitive impairment post-stroke is reported in up to 60% of ischemic stroke survivors ^2^ with varying incidence rates between 20–80% of individuals post-stroke ^3– 5^. A qualitative study involving 142 individuals post-stroke analysed data from follow-up assessment of functional outcomes and found that over half of individuals post-stroke who exhibit favourable recovery from physical deficits continue to experience cognitive deficits in the longer term ^6^. Indeed, cognitive deficits post-stroke are seen in 22% of individuals at 5 years post-stroke and 21% of individuals at 14 years post-stroke ^7^. Cognitive impairment post-stroke is shown to be independently associated with a lower quality of life ^8^, higher rates of mortality and institutionalisation ^9^, increased carer burden ^10^ and increased healthcare costs ^11^.

A priority setting partnership in the UK, the James Lind Alliance, identified that cognitive impairment post-stroke was the leading priority among the top 10 research priorities in relation to life after stroke ^12^. Despite this, much rehabilitation focus is placed on the improvement of physical deficits post-stroke, with a neglect towards cognitive deficits ^13, 14^. In their updated review examining the effects of physical fitness training post-stroke, Saunders *et al.* ^15^ noted that outcomes of cognitive function in particular lack investigation. Peoples *et al.* ^16^ reported from their synthesis of qualitative studies that people post-stroke report a focus during rehabilitation on physical needs over social re-integration and psychological support. These non-physical needs were perceived as factors that could facilitate empowerment and enable people post-stroke to regain control over their everyday life ^16^. Similarly, McKevitt *et al.* ^17^ estimated the prevalence of self-reported unmet needs in community-dwelling stroke survivors (n=799) across the United Kingdom and found that 60% of those surveyed reported memory problems after stroke as an unmet need. The lack of focus on cognitive deficits is further acknowledged by the Intercollegiate Stroke Working Party national clinical guidelines which highlight gaps in cognitive rehabilitation after stroke ^18^.

Cognition is not a unitary concept, as evidenced by the variety and breadth of neuropsychological assessments available ^19^. Cognitive impairment post-stroke involves a variety of deficits across multiple domains of cognition function ^20^ which allow an individual to select and process information within their environment ^21^. Cognitive rehabilitation is defined as "a systematic functionally orientated intervention of therapeutic cognitive activities based on the assessment and understanding of the patient's brain behaviour deficits" (Cicerone *et al.* ( 21, p.1596). Beyond the definition of specific “cognitive rehabilitation” interventions, a breadth of interventions can affect cognitive function in people post-stroke ranging from virtual reality training ^22^ to physical activity interventions ^23^ to neurofeedback therapy ^24^ and many more.

Living with memory deficits may result in negative effects on the stroke survivor and their family once in the community ^25^. A recent systematic review and meta-ethnography found that stroke survivors and carers can feel abandoned as a result of becoming marginalised by healthcare services ^26^. This marginalisation occurs due to lack of continuity of care, limitations in access to services and inadequate information provision to re-engage with services during different stages of recovery post-stroke. The lack of follow-up services was also noted ^26^. Specifically, people with memory problems post-stroke and their carers have identified barriers that may prevent them engaging with a healthcare professional such as fear of a dementia diagnosis, the denial of cognitive deficits and the lack of familiarity with HCPs to comfortably discuss their memory problems ^27^. These perceived unmet needs and inequities in accessing rehabilitation services are challenges that require attention. Moreover, given that research focused on the improvement of cognitive impairment post-stroke is considered as the top research priority among people post-stroke, carers and healthcare professionals, the design of an effective intervention and feasible intervention is an urgent issue.

The Medical Research Council’s (MRC) guidelines for developing complex interventions details the importance of identifying the current evidence base ^28^. To this end, a systematic review and meta-analysis was conducted by O’Donoghue *et al.* ^29^ to examine the totality of evidence with regard to interventions which rehabilitate cognitive deficits post-stroke. This review identified 64 studies and an extensive range of interventions including multiple component interventions, physical activity interventions, cognitive rehabilitation interventions, non-invasive brain stimulation (NIBS) protocols and occupational-based interventions. The protocol for this review has been published ^29^ and the review is at the write-up stage.

Given the breadth of potential approaches to cognitive rehabilitation identified, it is imperative to explore the insights of key stakeholders regarding their engagement with such interventions and their perceived effectiveness. The identification and engagement of stakeholders is essential to the development of a cohesive stroke system of care ^30^. Moreover, qualitative research methods have been proposed as key components in the conduct of research into complex interventions by increasing knowledge of intervention components and mechanisms of action ^31, 32^. To this end, the aim of this study is to examine the perspectives of key stakeholders on the design and delivery of an optimal intervention to rehabilitate cognitive deficits post-stroke.

## Methods

### Study design

This study will employ a qualitative descriptive approach with thematic analysis of data ^33, 34^. A qualitative descriptive approach was chosen so that broad and rich information would be gathered in relation to descriptions of participants' attitudes towards the development of a complex intervention ^32^. The study will be reported in line with the Consolidated Criteria for Reporting Qualitative Research (COREQ) checklist ^35^. The perspectives of key stakeholders will be gathered using a semi-structured interview protocol via telephone or telecommunication platform such as Microsoft Teams. 

### Research team roles

All interviews will be conducted, transcribed and analysed by the researcher (MOD), a physiotherapist and a PhD candidate at the University of Limerick. MOD has completed training in a qualitative research methodology module and also completed a training workshop for Nvivo software. Dr Sara Hayes (SH) is a lecturer in physiotherapy and an experienced quantitative and qualitative researcher. As the principal investigator, SH has led the conceptualisation of this research and will contribute to the data analysis and write-up of the manuscript. Dr Pauline Boland (PB) is an occupational therapist and an experienced qualitative researcher. Dr Rose Galvin (RG) is a senior lecturer in physiotherapy and experienced researcher in quantitative and qualitative research. Dr Siobhan Leahy is a lecturer in physiotherapy and an experienced quantitative and qualitative researcher. PB, RG and SL will contribute and provide critical feedback throughout the analysis, write-up and dissemination stages of this research. A third of the transcripts will be analysed by co-authors (SH, PB, SL) for peer coding and collaborative framework development. Another co-author (RG) will contribute to the clarification of themes at a later stage in the analysis

### Sampling and recruitment

A pragmatic mixed purposive sampling technique will be used in this study. Participants will include key stakeholder groups: people post-stroke, carers, healthcare professionals (HCPs), academics and academicians with an interest in stroke rehabilitation. Purposive sampling and snowball sampling will be used to recruit stakeholders ^36, 37^.

For people post-stroke, invitation letters and participant information leaflets will be sent via a gatekeeper from Volunteer Stroke Scheme and Headway in Limerick respectively. Both organisations are the key services supporting people post-stroke outside of health services. People post-stroke will be included if they meet the following inclusion criteria:

•   Aged 18 years or older with a diagnosis of ischaemic or haemorrhagic stroke
•   Self-reported cognitive problems post-stroke
•   In the acute, subacute or chronic stage post-stroke
•   Have the ability to verbally communicate over the phone/ via telecommunication platform.*
*In cases where an individual has difficulty understanding the interview questions, a proxy respondent may be used. The proxy respondent may be a spouse or a carer of the individual post-stroke
People post-stroke will be excluded if:
•   Individuals are unable to provide informed consent. Ability to provide consent will be determined at eligibility checking stage though the gatekeeper and/or proxy respondent.

Carers will include caregivers, spouses or family members who provide care (paid or unpaid), support or assistance to people post-stroke ^38^. Invitation letters and participant information leaflets will be sent through a gatekeeper from The Carers Association Limerick, a local carers’ branch in the Limerick area (see extended data ^39^). Carers of eligible people post-stroke are also eligible for inclusion. Carers will be eligible for inclusion if they have been involved/ are currently involved in the care of an individual post-stroke.

HCPs will be recruited from relevant neurological clinical sites i.e. those working in stroke units of acute hospitals, rehabilitation units of subacute hospitals or community settings, and Twitter. HCPs will be eligible for inclusion if they have treated or are treating people post-stroke. HCPs will include medical staff, physiotherapists (PTs), occupational therapists (OTs) psychologists and speech and language therapists (SLTs) working in the provision stroke rehabilitation services. Academics and academicians will include those affiliated with the University of Limerick with an interest in stroke rehabilitation research who will be invited through relevant research clusters within the University of Limerick such as the Ageing Research Cluster (ARC) and the Physical Activity for Health Research Cluster (PAfH).

Once prospective participants express an interest in engaging with the study, a phone call will be scheduled to discuss what participation entails, ensuring that the potential participant has read and understood the participant information leaflet already supplied. The participant will be required to complete a consent form and a brief demographic information form prior to their interview/focus group (see extended data ^39^). The researcher will orally present the participant information leaflet and offer to email/ post it. The researcher will arrange a follow-up call a minimum of one week later to allow time to consider participation in the study. During the follow-up call, if the individual wishes to participate, verbal informed consent will be confirmed orally and audio recorded. Alternatively, if the individual wishes to read the participation leaflet and sign the consent form themselves, the researcher will post the participant information leaflet and informed consent form to the individual (using a pre-paid postage stamped letter; see extended data ^39^). The individuals will then send the signed consent form back to MOD. Completion of the written informed consent form, either by written or digitally signed signature, will be a prerequisite for participation in the study in accordance with the Health Research Regulations (2018) ^40^.

Participation in interviews and/or focus groups will only commence once informed consent from all participants is received. According to the Assisted Decision-Making Capacity Act (2015) ^41^, individuals should be assumed to have the capacity to consent unless otherwise demonstrated. A key issue in the context of this study is the manner in which the capacity for informed consent is established and how potential changes in capacity are identified. To this end, the capacity of participants with cognitive impairment post-stroke to consent to participate in the study will be assessed on a continual basis, with regular re-iteration of the rights of participants as the study progresses.

The impact of cognitive deficits post-stroke on decision-making around participation in research must be considered ^42^. For this reason, informed consent will be conducted as an ongoing process i.e., where consent will be obtained prior to participation, reviewed on the day of participation and reviewed again after participation has occurred. Moreover, the content of all consent forms will be discussed with participants in line with best practice to ensure participant understanding of the nature of their participation in this study and any risks associated with same (Lewis and Graham 2007). MOD will refer to the INVOLVE guidelines regarding the knowledge, skills and experience required to participate in PPI when addressing participant queries ^43^.

### Sample size

The sample size for this study will follow guidance from Malterud *et al.* (2016) regarding “information power.” According to this model, criteria such as the aim of the study, the specificity of the sample of participants, the use of established theory, the quality of dialogue and the analysis strategy should ascertain whether sufficient information power will be achieved with less or more participants included in the study sample ^44^. In the context of this study, “information power” appears more suitable than “data saturation” in the process of decision-making regarding sample sizes, given that the concept of data saturation is often poorly defined within qualitative studies and the methods by which authors claim data saturation are not always transparent ^45– 47^. In accordance with this model and in consideration of the current study, it is anticipated that approximately ten interviews will be conducted with individuals post-stroke, ten interviews with caregivers and ten interviews with healthcare professionals.

### Data collection

The qualitative research interview is a highly utilised data collection tool in health research ^48^. The most common type of interview used in qualitative research and the healthcare context is the semi-structured interview which enables an in-depth exploration into the experiences of interviewees and offers the insights into how difference phenomena of interest are perceived ^49, 50^. This study will employ both telephone-based and online methods of data collection, wherein face to face contact may not be possible due to COVID-19.

Semi-structured interviews and focus groups will be conducted using a semi-structured interview protocol (see extended data ^39^) via telephone or via a telecommunication platform such as Microsoft Teams, as per the participants’ preferences and in accordance with the tools that are licensed and supported by ITD, University of Limerick.

The telephone interview is an effective method for collection of qualitative data which facilitates flexible interview scheduling, less time-consuming for both the researcher and participant, enhanced access to geographically dispersed regions and more cost-effective ^51^. Furthermore, telephone interviewing can mitigate against some of the negative aspects of face-to-face interviewing; it is argued that telephone-based interviews offer a more balanced distribution of power between researcher and participant and offer a greater level of anonymity and privacy than with face-to face interviews ^52, 53^. 

### Proxy respondents

In cases where the individual post-stroke is unable to effectively communicate in a semi-structured interview setting, proxy respondents may be used. Given that post-stroke aphasia occurs in up to 42% of people post-stroke in the acute setting and up to 50% in rehabilitation or community settings ^54^, it is important that these individuals are empowered to express their views ^55^. However, proxy ratings must be interpreted with caution given that proxy respondents are likely to overestimate the level of impairment of the individual, compared to self-reported measures ^56^. Therefore, all summaries of data collected by the proxy respondent will be summarised and relayed back to the individual post-stroke who may wish to add their views to the reported carer data. 

As well as the use of proxy respondents, this study will employ a number of adjustments to the qualitative interviewing skill in order to be as facilitating and inclusive as possible. Strategies such as reducing the cognitive load on individuals by lessening the content of the interview line of questioning and utilising clear and visual forms of communication where possible will be used ^57^. Furthermore, people post-stroke will be interviewed remotely while situated in their home to provide a familiar and relaxed environment to facilitate open communication during the interview process and in which individuals are more likely to disclose information relating to the nature of their lived experiences ^58^. A focus group may be conducted with carers or HCPs who work on the same clinical site. Where a focus group is not possible, individual interviews will be conducted with these stakeholders.

### Topic guides

The interview topic guide and questions were developed by the research team by reviewing existing literature regarding the engagement with rehabilitation services post-stroke. The interview questions were based on the principles of developing semi-structured interviews in qualitative research and health research respectively ^48, 59^. The template for intervention description and replication checklist for reporting of interventions (TIDieR) was used to frame questions relating to the design and delivery of a future intervention to rehabilitate cognitive deficits post-stroke ^60^. The interview guides for each stakeholders group are available as extended data ^39^.

### Piloting

A piloting phase will be undertaken to assess the acceptability of the semi-structured interview for stakeholders. An individual from each stakeholder group will be asked to provide feedback on the flow of questions, the relevance of questions and their overall experience of the interview process focusing on emotional impact and feelings of fatigue or overload. Following this, these individuals will participate in cognitive interviews centred on their experience of completing the semi-structured interview. Cognitive interviewing is a pre-testing strategy that explores how respondents interpret and attribute meaning to individual questions ^61, 62^. It can identify issues regarding the terminology of questions which could lead to potential misinterpretations, resulting in incomparable responses and missing data ^61^. While this strategy is primarily used in questionnaire design ^61^, it was deemed useful in this qualitative interview study as, given the likely age, health status, cognitive and communication difficulties, there is a risk of misinterpretation of interview questions associated with stakeholders in this study. The following techniques guide cognitive interviewing: think/ read aloud, cognitive verbal probing and observation ^61^. Given that respondents will have completed the pilot interview, it is important to consider the cognitive load associated with the subsequent cognitive interview. Therefore, cognitive verbal probing will be used as the main approach to guide the cognitive interview and will be guided using a brief list of prompts. 

### Data protection

All data will be handled confidentially and will be stored in accordance with the Data Protection Policy at the University of Limerick. All consent forms will be stored in a locked cabinet in the Principle Investigator’s office in the School of Allied Health Building, University of Limerick. Participants will be assigned a unique participant number when data are transcribed. A separate, password-protected Excel file will hold participants' details and their unique participant number on a password protected laptop. Audio files will be destroyed after being transcribed and the research team will only have access to anonymised transcripts. These transcripts and descriptive statistics will be stored on a password-protected laptop.

### Data analysis

Nvivo software package (Version 12 QSR International) will be used to import transcripts, organise and retrieve data to be analysed. Data will be analysed and collectively synthesised using reflexive thematic analysis ^63^. Reflexive thematic analysis was chosen for its theoretical flexibility as well as its ability to provide a rich and detailed account of a large dataset ^63, 64^ and is a useful approach for evaluating the perspective of different research participants, highlighting the similarities and difference between groups and generating a rich and detailed account of the data ^65^. Data will be analysed though an iterative process where data collection and data analysis will occur concurrently and recursively to integrate the development of themes grounded in the primary data ^66^. The flexibility of this approach, while useful, also has the potential to lead to inconsistencies in the development of themes derived from the data ^67^. To reduce this risk, this study will adhere to standardised criteria promoting trustworthiness as outlined by Nowell *et al.* (2017). This step by step procedure aims to guide qualitative researchers to meet the original trustworthiness criteria outlined by Lincoln and Guba (1985). Data will be analysed using an inductive approach, where generated codes will be based on the content of the primary data rather than existing theories or concepts.

Reflexive thematic analysis will be conducted in accordance with the steps outlined by Braun and Clarke (2019) which will be applied to the current study: (i) data familiarisation, involving repeated active engagement with notes and transcripts. Initial theoretical and reflexive thoughts will be documented to inform the next step, (ii) generation of initial codes; all segments of data which may be relevant to the research question will be coded, (iii) conceptualisation of themes, involving the identification and interpretative analysis of the collated codes (iv) reviewing themes, involving the refinement of themes identified. This will require in-depth interpretation and reviewing of the boundaries of each theme and probing to decipher if there is sufficient data to support the theme. There should be a clear and identifiable distinction between themes and data within these themes should cohere in a meaningful manner (v) defining and naming of themes, requiring clear and descriptive working definitions to be generated for each theme and potential subthemes within the data, (vi) producing the final report, involving the transformation of the analysis into the publication of a journal article.

### Ethics approval

Ethical approval for this study has been granted by the Faculty of Education and Health Sciences Research Ethics Committee at the University of Limerick [Ref 2020_03_05_EHS].

### Dissemination

Study findings will be submitted for publication in peer-reviewed journals and will be disseminated through relevant research clusters in the University of Limerick. We will engage with participating networks such as the Volunteer Stroke Scheme, Headway Limerick and University Hospital Limerick to disseminate the results of our study. This will be done through a public talk in the community involving all stakeholders and their families. Abstracts will be submitted to relevant national and international conferences. Findings will also be disseminated through the use of social media such as Twitter.

### Study status

Recruitment and data collection for this study commenced in November 2020. 

## Conclusion

In accordance with the MRC framework for developing and evaluating complex interventions, the findings from this study will be combined with findings from a quantitative systematic review exploring the evidence in support of interventions to rehabilitate cognitive deficits post-stroke. This combined evidence base will result in impactful, user-informed research and will inform the design of an intervention protocol to rehabilitate cognitive deficits post-stroke.

## Data availability

### Underlying data

No data is associated with this article.

### Extended data

Figshare: Exploring the perspectives of people post-stroke, carers and healthcare professionals to inform the development of an intervention to improve cognitive impairment post-stroke. https://doi.org/10.6084/m9.figshare.13332923.v1 ^39^

This project contains the following extended data:

- Appendices.docx (recruitment letters/emails, participant information leaflets, consent forms and demographic data collection form
- Interview guide.docx (cognitive interview guide and semi-structured interview guides for individuals post-stroke, carers and healthcare professionals)

Data are available under the terms of the Creative Commons Attribution 4.0 International license (CC-BY 4.0).
