# Supplementary material for: Exploring the perspectives of key stakeholders on the design and delivery of an intervention to rehabilitate people with cognitive deficits post-stroke
Source: HRB Open Res. 2021 Jul 8;3:93. Originally published 2020 Dec 22. [Version 2] doi: 10.12688/hrbopenres.13184.2 (PMC10879761; doi:10.12688/hrbopenres.13184.2)
Supplement: Peer review history

# Reviewer report: Reviewer response for version 2

**DOI:** 10.21956/hrbopenres.14544.r38193

**Published:** 2024-02-20

**Authors:** Salvatore Facciorusso

**Affiliations:** 1 Villa Beretta Rehabilitation Center, Universita degli Studi della Campania Luigi Vanvitelli, Caserta, Campania, Italy

The study protocol provided aims to explore the perspectives of key stakeholders on the design and delivery of an intervention to rehabilitate people with cognitive deficits post-stroke. However, i have some concerns.

1. The major concern: Considering the study was initially designed under the constraints of COVID-19 restrictions, which influenced its methodological choices, it is now advisable to adapt the study protocol to reflect the current context where these restrictions have been eased. You wrote "This study will employ both telephone-based and online methods of data collection, wherein face to face contact may not be possible due to COVID-19."Therefore, it would be prudent to revisit and modify the methods accordingly. Below are some recommendations for adaptation::
  - Consider adding face-to-face interviews or focus groups to capture richer data, including non-verbal cues.
  - Develop protocols for in-person, remote or hybrid interactions to ensure the safety and comfort of participants, offering them the choice of communication mode.
  - The changes in data collection methods and recruitment strategies might necessitate adjustments to the project's feasibility and timeline.
  - Allocate resources for logistical and technological support required for face-to-face data collection.
  - **Data Collection**:The choice of telephone and online interviews, while practical in the context of COVID-19 restrictions, may limit the depth of interactions compared to face-to-face interviews. Non-verbal cues are lost, which can be particularly relevant when discussing sensitive topics like cognitive impairments post-stroke. COVID 19 restrictions are now limited. Thus, If feasible, offer participants the option of face-to-face interviews in addition to telephone and online interviews. This would accommodate participants' preferences and potentially enhance the depth of qualitative data collected. The reliance on technology may exclude participants who are not comfortable with or do not have access to the necessary tools. 
  - **Proxy Respondents**:The use of proxy respondents introduces another layer of complexity, as proxies may not accurately represent the experiences and perspectives of the stroke survivors themselves. This methodological choice could affect the validity of the data collected, especially considering the potential overestimation of impairments by proxies compared to self-reports. Whenever possible, include direct assessments or observations of stroke survivors alongside proxy reports. Offer guidance for proxy respondents on how to accurately represent the experiences and perspectives of the stroke survivors they are speaking for.

Finally, In the context of your study, the utilization of teleconsultation methods has proven to be an invaluable tool, especially given the constraints imposed by the COVID-19 pandemic in these years. However, with the progressive easing of these restrictions, it presents an opportune moment to reassess and update your study protocol to reflect the current landscape and emerging needs.

Is the study design appropriate for the research question?

Partly

Is the rationale for, and objectives of, the study clearly described?

Yes

Are sufficient details of the methods provided to allow replication by others?

Partly

Are the datasets clearly presented in a useable and accessible format?

Not applicable

Reviewer Expertise:

Stroke rehabilitation

I confirm that I have read this submission and believe that I have an appropriate level of expertise to confirm that it is of an acceptable scientific standard, however I have significant reservations, as outlined above.

# Reviewer report: Reviewer response for version 2

**DOI:** 10.21956/hrbopenres.14544.r29783

**Published:** 2021-07-12

**Authors:** Sinéad Hynes

**Affiliations:** 1 Discipline of Occupational Therapy, National University of Ireland, Galway, Ireland

Thank you to the authors for clarifying the questions I had and for adding additional detail in areas that were requested. 

 There is one typographical error in heading that should read "Focus groups".

Is the study design appropriate for the research question?

Partly

Is the rationale for, and objectives of, the study clearly described?

Partly

Are sufficient details of the methods provided to allow replication by others?

Yes

Are the datasets clearly presented in a useable and accessible format?

Not applicable

Reviewer Expertise:

Cognitive rehabilitation, community-based rehabilitation, intervention development.

I confirm that I have read this submission and believe that I have an appropriate level of expertise to confirm that it is of an acceptable scientific standard.

# Reviewer report: Reviewer response for version 1

**DOI:** 10.21956/hrbopenres.14316.r28577

**Published:** 2021-01-27

**Authors:** Margaret McGrath

**Affiliations:** 1 Sydney School of Health Sciences, The University of Sydney, Sydney, NSW, Australia

This qualitative study sets out to explore experiences of post-stroke cognitive impairment and perspectives on the optimum approach to cognitive rehabilitation after stroke from the perspective of stroke survivors, carers of stroke survivors and health care professionals working in stroke rehabilitation. 

 The authors suggest that the findings of the study, when combined with findings from a systematic review of interventions to address post stroke cognitive impairment will 'inform the design and development of an intervention to rehabilitate cognitive impairment post stroke'. 

 I have noted below some points for consideration by the authors. 

**Rationale and objectives:**

- The authors present a compelling case for the research. The research is complemented by an earlier systematic review of interventions for post stroke cognitive impairment. 
- There are two different research aims and it would be helpful for the authors to set out these aims more explicitly before addressing the research design - one to describe the experience of post stroke cognitive impairment from the perspective of the stroke survivor/care and health care professionals. A second aim is to explore these stakeholders views on interventions to address cognitive impairment. 
- There are three distinct groups of potential participants: stroke survivors; carers of stroke survivors and health care professionals. The inclusion of multiple perspectives is helpful given the complexity of post stroke cognitive impairment. 
- An estimated 10 stroke survivors will be recruited to the study. The authors refer to purposive sampling but it is not clear how this will be enacted - beyond fulfilling the inclusion criteria how will participants be selected? 
- I am not entirely clear how the authors propose to include the perspectives of people with communication impairment - it is likely that using supportive communication strategies participation of this cohort could be facilitated rather than relying on a proxy respondent. It may be useful to draw on the expertise of a speech and language pathologist to support this. 
- Given that many stroke survivors/carers have unmet needs relating to cognitive impairment I am not clear how they will be expected to give informed answers about their preferences for intervention approaches, timing or delivery methods. It seems more likely that their opinions will be based on a hypothetical 'if services were available' and this limitation should be acknowledged. 

**Health professionals:**

- It seems as if most of the health professionals will be recruited from a local health service and it is not clear if the health professionals will have direct clinical experience in addressing cognitive impairment post stroke. It may be worth considering expanding the scope of potential recruitment to address this gap. 
- It is not clear why you have chosen not to include nursing staff among HCP to be recruited?

**Data collection:**

- It would be helpful for the reader to clarify which participants will be offered the opportunity to engage in a focus group and which participants will be interviewed. 
- The process for data analysis is clear and comprehensive. 

**General comments: **

- The methods section would benefit from further editing as at times information is repeated. 

Is the study design appropriate for the research question?

Yes

Is the rationale for, and objectives of, the study clearly described?

Partly

Are sufficient details of the methods provided to allow replication by others?

Partly

Are the datasets clearly presented in a useable and accessible format?

Yes

Reviewer Expertise:

Occupational Therapy; Neurorehabiltiation; Chronic Disease and Disability;

I confirm that I have read this submission and believe that I have an appropriate level of expertise to confirm that it is of an acceptable scientific standard, however I have significant reservations, as outlined above.

# Response

**Published:** 2021-03-31

**Authors:** mairead o donoghue

**Affiliations:** University of Limerick, Ireland

Many thanks to the reviewers for their comprehensive feedback and comments on this protocol paper. We really value this feedback. As we are currently submitting a review paper that informs this qualitative work, we have decided to delay our response to reviewer comments for a month or so. We will respond in full after this time. Thank you again for your valuable feedback.

 Kind regards,

 Mairéad O' Donoghue.

# Response

**Published:** 2021-07-02

**Authors:** mairead o donoghue

**Affiliations:** University of Limerick, Ireland

Thank you for taking the time to review this protocol. I apologise for the delay in response. 

**Point 1: **The authors present a compelling case for the research. The research is complemented by an earlier systematic review of interventions for post stroke cognitive impairment. 

**Response 1: **Thank you. 

 Point 2: There are two different research aims and it would be helpful for the authors to set out these aims more explicitly before addressing the research design - one to describe the experience of post stroke cognitive impairment from the perspective of the stroke survivor/care and health care professionals. A second aim is to explore these stakeholders views on interventions to address cognitive impairment.

**​​​​​​​Response 2: **Thank you for highlighting this. We agree that the research aim needs to be set out more explicitly. To this end, and in accordance with changes to the manuscript title, the manuscript has been amended to reflect one specific aim of this study, “to elicit the perspectives of key stakeholders on the design and delivery of an intervention to rehabilitate cognitive deficits post-stroke.” Please see Introduction section Line 149-151. 

**Point 3: **Rationale and objectives:

- There are three distinct groups of potential participants: stroke survivors; carers of stroke survivors and health care professionals. The inclusion of multiple perspectives is helpful given the complexity of post stroke cognitive impairment. An estimated 10 stroke survivors will be recruited to the study. The authors refer to purposive sampling but it is not clear how this will be enacted - beyond fulfilling the inclusion criteria how will participants be selected? 

**​​​​​​​Response 3: **Thank you for requesting clarity on this. We have added a sentence to outline how we will work with gatekeepers of organisations supporting people post-stroke to facilitate purposive sampling. Please see Methods, subheading: Sampling and recruitment, Line 200-201;

**Point 4: **I am not entirely clear how the authors propose to include the perspectives of people with communication impairment - it is likely that using supportive communication strategies participation of this cohort could be facilitated rather than relying on a proxy respondent. It may be useful to draw on the expertise of a speech and language pathologist to support this. 

**​​​​​​​Response 4: **Thank you for requesting clarity on this. We have added to the manuscript outlining supportive communication strategies that will be used in order to be as inclusive as possible. Any issues that do arise regarding cognitive communication difficulties will be factored in the feasibility study. Please see Methods section; Topic Guides and Piloting, Line 372-377.

**Point 5: **Given that many stroke survivors/carers have unmet needs relating to cognitive impairment I am not clear how they will be expected to give informed answers about their preferences for intervention approaches, timing or delivery methods. It seems more likely that their opinions will be based on a hypothetical 'if services were available' and this limitation should be acknowledged. 

**​​​​​​​Response 5: **Thank you. A similar comment was raised by both reviewers regarding the “hypothetical, ideal world” situation versus “real world” situation. Yes, we agree that stakeholders may have unmet needs relating to cognitive impairment. We aim to illicit their views on the gaps in the services received and their experience of cognitive intervention to date i.e. the “real world” experience. We also hope to garner perspectives on the design of a “hypothetical, optimal” intervention in line with the “who, what, where, when and why” line of questioning of the TIDER framework (Hoffmann *et al.* 2014). We will acknowledge opinions based on a hypothetical view as a limitation in the main results study.

**Point 6: **Health professionals:

- It seems as if most of the health professionals will be recruited from a local health service and it is not clear if the health professionals will have direct clinical experience in addressing cognitive impairment post stroke. It may be worth considering expanding the scope of potential recruitment to address this gap. 

**​​​​​​​Response 6: **Thank you for this comment. One means of recruiting health professionals is from a local service, and another is through social media (Twitter) with no limit on geographical location. Given the use of Twitter as a platform to advertise our study, and that data will be collected online due to Covid-19 restrictions, we foresee recruitment extending far beyond local health services.

 We have edited the inclusion criteria for health professionals to outline that health professionals must have experience in the treatment of individuals post-stroke with cognitive impairment in order to be eligible. Please see Methods, subheading: Sampling and recruitment. 

**Point 7: **It is not clear why you have chosen not to include nursing staff among HCP to be recruited?

**​​​​​​​Response 7: **Thank you for this question. We aimed to include HCPs most likely to delivery such an intervention but we recognise that some were not included e.g. nursing staff. This will be acknowledged as a limitation in the results manuscript.  

**Point 8: **Data collection:

 It would be helpful for the reader to clarify which participants will be offered the opportunity to engage in a focus group and which participants will be interviewed. 

**​​​​​​​Response 8: **HCPs alone, if working on the same clinical site and if deemed more feasible to facilitate, will be offered to engage in a focus group. People post-stroke and caregivers will be interviewed. This has been clarified in the Methods, subheading: proxy respondents. 

**Point 9: **The process for data analysis is clear and comprehensive. 

**​​​​​​​Response 9: **Thank you.

# Reviewer report: Reviewer response for version 1

**DOI:** 10.21956/hrbopenres.14316.r28578

**Published:** 2021-01-18

**Authors:** Sinéad Hynes

**Affiliations:** 1 Discipline of Occupational Therapy, National University of Ireland, Galway, Ireland

**Summary:**

 This protocol of a qualitative study examines the perspectives of key stakeholders on cognitive rehabilitation for people post-stroke. Through a series of interviews (with people who have had a stroke and are experiencing cognitive difficulties, healthcare professionals, carers, and academics), the authors aim to gather data that will be used to inform the development of a cognitive rehabilitation intervention for people post-stroke. The protocol provides a good level of detail, including supporting material, that allows for replication of the research. The authors make reference to the use of the template for intervention description and replication checklist for reporting of interventions (TIDieR) and The Consolidated Criteria for Reporting Qualitative Studies (COREQ) guidelines which are good to see in the development of the research protocol. There are a number of areas that I think need clarification or some additional detail in order for the protocol to be complete. 

**Aim:**

 Given that the title states that the study will “ *inform the development of an intervention to improve cognitive impairment post-stroke*.” there is not adequate discussion with regards to how the results of this research will be used to inform an intervention.  

 The research aim in the manuscript is a better match with the focus of what is proposed:

 “ *The aim of this study is to examine the perspectives of key stakeholders on the design and delivery of an optimal intervention to rehabilitate cognitive deficits post-stroke*.”

**Background:**

 There has been much done in this field in the past- how will previous research be integrated into the planned intervention? The background should provide more detail on cognitive rehabilitation interventions that have a promising/proven evidence base. There is reference to a not-yet published review on the topic which is not sufficient, given the aim of intervention development.

 It would be useful to have a stronger rationale for the current study, given that there is an extensive literature base in the area. What was the rationale for focusing on stakeholder views on intervention? Is the specific focus here on the feasibility in practice or is it something else- it would be good to see this clarified. Given the broad range of cognitive deficits seen in people post-stroke, will the proposed cognitive intervention focus on all areas proposed here or will it be specific to an area- e.g. memory, neglect, etc. Some clarification would be beneficial for the reader.

**Eligibility:**

*People post-stroke*- How will the inclusion criteria of “self-reported cognitive problems” be met?

 What are the limitations of not including people post-stroke who cannot provide informed consent?

 With regards to “changes to capacity” and this being “assessed on a continual basis” and “reviewed again after participation has occurred”- is it not a one-off involvement for participants? Why is there a need for monitoring of capacity in this study?

*Proxy respondent*- will they be assisting the person post-stroke to answer the questions or giving their own responses? When and how will the decision be made to use a proxy? Would be good to get more detail of what this would look like in practice- will the person post-stroke be present for the interview?

*Caregiver criteria*- is there a minimum amount of care that needs to be provided by carers for them to be deemed eligible? “Have been involved in care”- does it matter how long ago this might be? Given that data is being collected remotely had you thought about recruiting from outside of Limerick?

*Academics*- How will the authors deal with a potential conflict of interest or coercion when recruiting researchers from their own research group to participate in the study? How might this affect the data collected?

**Data collection:**

 There are a number of references to focus groups in the manuscript- “semi-structured interviews and focus groups”.  The rationale and methodology for focus groups and interviews is distinct. Need to see a clearer rationale for the use of interviews/focus groups with different groups in the study. Privacy and anonymity are mentioned (column 2, top of page 5) and this would not be guaranteed with focus groups.

**Interview guide**:

*Participants post-stroke*

- Interview guide- questions are very specific for someone with cognitive difficulties- e.g. exact details of materials used in previous rehab programmes.
- In the “what” section – are participants asked about each of these intervention types? Are they Yes/no responses? It would be good to have more clarification here as there is a danger of leading participants through some of the suggestions. 
- Some of the questions are quite complicated.
- Would the participant be expected to know who would best deliver the intervention?

How long on average are the interviews? Seems quite a substantial amount of material for someone with cognitive difficulty (and potential cognitive fatigue).

 Is the same guide used for focus groups?

*Guide for HCPs*- are you interested in “the ideal world” or what is feasible in their own clinical practice?

**Data analysis:**

 How will the separate data sets be integrated? Will the data be analysed separately for each of the groups or will this be integrated? Will interview and focus group data be analysed in the same way? Will member-checking only be used with proxy interviews? There is reference to "trustworthiness" but not adequate detail in relation to how this will be ensured. 

**Conclusion**

 How exactly will the findings be used to inform an intervention? This needs to be clearer given the title and aim of the research. The impact and need for the research should be clear.

Is the study design appropriate for the research question?

Partly

Is the rationale for, and objectives of, the study clearly described?

Partly

Are sufficient details of the methods provided to allow replication by others?

Yes

Are the datasets clearly presented in a useable and accessible format?

Not applicable

Reviewer Expertise:

Cognitive rehabilitation, community-based rehabilitation, intervention development.

I confirm that I have read this submission and believe that I have an appropriate level of expertise to confirm that it is of an acceptable scientific standard, however I have significant reservations, as outlined above.

# Response

**Published:** 2021-07-02

**Authors:** mairead o donoghue

**Affiliations:** University of Limerick, Ireland

Thank you for reviewing this protocol. I apologise for the delay in response. 

**Point 1:** Given that the title states that the study will “ *inform the development of an intervention to improve cognitive impairment post-stroke*.” There is not adequate discussion with regards to how the results of this research will be used to inform an intervention. The research aim in the manuscript is a better match with the focus of what is proposed:

 “ *The aim of this study is to examine the perspectives of key stakeholders on the design and delivery of an optimal intervention to rehabilitate cognitive deficits post-stroke.*

**Response 1:** Thank you for this valuable feedback. We have added further discussion outlining how stakeholder perspectives will inform the development of an intervention to improve cognitive impatient post-stroke in more detail. Please see Introduction section, Line 142-145, Line 147-156. We have also changed the title of this manuscript to reflect a clearer message of the study. 

**Point 2:** Background:

 There has been much done in this field in the past- how will previous research be integrated into the planned intervention? The background should provide more detail on cognitive rehabilitation interventions that have a promising/proven evidence base. There is reference to a not-yet published review on the topic which is not sufficient, given the aim of intervention development.

**Response 2:** Thank you for this question. We have added additional text and referenced additional sources to outline previous research on cognitive rehabilitation interventions. We have added evidence from previous Cochrane reviews. Please see Introduction, Line 93-105.  We have also expanded on the findings of our quantitative systematic review. Please see Introduction, Line 116-119. The referenced systematic review protocol is for a systematic review which is currently under review with *Stroke AHA*. We hope to hear a decision on this soon. For now, we have referenced the published systematic review protocol.

**Point 3:** It would be useful to have a stronger rationale for the current study, given that there is an extensive literature base in the area. What was the rationale for focusing on stakeholder views on intervention?

**Response 3: **Thank you. The rationale for focusing on stakeholder views is that we wish to elicit the perspectives of key stakeholders on the design and delivery of an intervention to rehabilitate cognitive deficits post-stroke. Such a starting point will aid early knowledge on feasibility and acceptability of future interventions. We have clarified this in the manuscript. Please see Introduction, Line 147-154.

**Point 4: **Is the specific focus here on the feasibility in practice or is it something else- it would be good to see this clarified

**Response 4: **The specific focus is to gain the perspectives of key stakeholders to inform the development phase of the MRC framework. In accordance with the MRC framework, perspectives of relevant stakeholders will be used to inform the development and design of an evidenced-based and stakeholder informed feasibility study rehabilitate cognitive deficits post-stroke. This has been clarified in the Introduction, Line 150-156.

**Point 5: **Given the broad range of cognitive deficits seen in people post-stroke, will the proposed cognitive intervention focus on all areas proposed here or will it be specific to an area- e.g. memory, neglect, etc. Some clarification would be beneficial for the reader.

**Response 5: **We agree that cognitive functioning is very diverse. The findings from this qualitative study will help determine what specific domain/ domain(s) of cognitive impairment our intervention will focus on, or indeed if a domain will be focused on. The content of the proposed intervention will be prioritised based on the findings of our previous quantitative systematic review, coupled with our future qualitative findings.

 We have added to the manuscript to outline items in the interview schedule which include questioning around a breadth of rehabilitation interventions and multiple domains of cognitive functioning. Please see Methods section, subheading: Topic Guides, Line 393-397.

**Point 6: **People post-stroke- How will the inclusion criteria of “self-reported cognitive problems” be met?

**Response 6: **The gatekeepers of both the Stroke Support Group and Headway are in a position to identify people with cognitive difficulties post-stroke and as such, will identify individuals that may be suitable to participate based on pre-defined eligibility criteria..

 Once potential participants have been identified, and have received and understood the participant information leaflet, the researcher will re-assess eligibility based on inclusion criteria as outlined in the manuscript. A line has been added to the Methods section to demonstrate the role of the researcher in confirming the eligibility of the potential participant during the recruitment process. This will entail the person with stroke confirming they have cognitive issues as a result of their stroke. Please see Methods, subheading: Sampling and Recruitment, Line 243-245.

**Point 7: **What are the limitations of not including people post-stroke who cannot provide informed consent?

**Response 7: **Thank you for requesting clarity on this. The inability to include people unable to consent, due to clear ethical concerns, means that their perspectives on intervention design will be missing from this study, which we will acknowledge as a limitation of this study.

 However, we have attempted to address this via the use of proxy respondents, by including the views of caregivers and reducing the cognitive load on people post-stroke using. Please see details of how we aim to facilitate people post-stroke during the interview in Methods, subheading: Proxy respondents.

**Point 8:** With regards to “changes to capacity” and this being “assessed on a continual basis” and “reviewed again after participation has occurred”- is it not a one-off involvement for participants? Why is there a need for monitoring of capacity in this study?

**Response 8: **Thank you for this question. “Reviewed again after participation has occurred” refers to immediately after the interview had been conducted. We are not following up at a later date. The monitoring of capacity is only relevant for people post-stroke. People post-stroke will be assessed for capacity to consent on a continual basis throughout the interview given that cognitive deficits may interfere with the decision making process regarding participation in research. We have clarified this in Methods, Line 273. We have also added that participants may withdraw from the study at any time. Please see Methods, subheading: Sampling and recruitment, Line 276-277. 

**Point 9:** *Proxy respondent*- will they be assisting the person post-stroke to answer the questions or giving their own responses? When and how will the decision be made to use a proxy? Would be good to get more detail of what this would look like in practice- will the person post-stroke be present for the interview?

**Response 9: **Thank you. We have clarified the role of proxy respondents in the manuscript. Please see Methods, subheading: proxy respondents, Line 327-331.  

**Point 10:** *Caregiver criteria*- is there a minimum amount of care that needs to be provided by carers for them to be deemed eligible? “Have been involved in care”- does it matter how long ago this might be?

**Response 10: **No, there is not a minimum amount of care that needs to be provided by carers for them to be deemed eligible. In accordance with the definition used to identify caregivers, “Carers will include caregivers, spouses or family members who provide care (paid or unpaid), support or assistance to people post-stroke (Greenwood *et al.* 2008)”. This has been clarified in the Methods, subheading: Sampling and recruitment, Line 220-221.

**Point 11:** Given that data is being collected remotely had you thought about recruiting from outside of Limerick?

**Response 11: **Yes, we had considered recruiting outside of Limerick. This study will use both purposive and snowball sampling of participants. Snowball sampling of participants would permit recruitment to extend beyond the Limerick area. The use of Twitter will also allow recruitment to extend beyond the Limerick area also. We have added to the manuscript outlining that participants will be recruited outside the Limerick area. Please see Methods, 193-195. 

**Point 12:** *Academics*- How will the authors deal with a potential conflict of interest or coercion when recruiting researchers from their own research group to participate in the study? How might this affect the data collected?

**Response 12: **We will acknowledge the potential relationship between the researchers and academicians recruited. We have added to Methods to outline how this potential conflict of interest will be managed.  Please see Methods, Sampling and Recruitment, Line 236-239.

**Point 13:** Data collection:

 There are a number of references to focus groups in the manuscript- “semi-structured interviews and focus groups”.  The rationale and methodology for focus groups and interviews is distinct. Need to see a clearer rationale for the use of interviews/focus groups with different groups in the study. Privacy and anonymity are mentioned (column 2, top of page 5) and this would not be guaranteed with focus groups.

**Response 13: **Thank you. We plan to conduct focus groups only for health professionals working on the same clinical site. Due to the remote nature of conducting our data collection due to Covid-19, people post-stroke and caregivers will be interviewed in their own homes and as such, a focus group will not be conducted with these groups. We have amended the text to clarify this. Please see Methods, subheading: proxy respondents, Line 349.  

 We agree that focus group methodology raises ethical challenges with regard to the preservation of privacy and anonymity. We have added to the manuscript to outline the means of addressing these issues. Please see Methods, subheading: Focus Groups, Line 350-358.

**Point 14:** Interview guide:

*Participants post-stroke*

- Interview guide- questions are very specific for someone with cognitive difficulties- e.g. exact details of materials used in previous rehab programmes.
- In the “what” section – are participants asked about each of these intervention types? Are they Yes/no responses? It would be good to have more clarification here as there is a danger of leading participants through some of the suggestions. 

**Response 14: **Thank you for requesting clarity on our interview guides. The “what” section of the interview would open up responses on the experience or knowledge of the individual regarding a particular intervention, if they are able to recall this. In some cases, specific intervention examples may be helpful to assist with gathering of information i.e. people with recall issues. If they are not able to recall these details, more general information will be gathered. These are suggested questions and not all are mandatory, the interviewer will adapt to the communication ability and preference of the interviewee on a case by case basis. If the participant wishes to answer in a yes/ no format, the researcher will probe using prompts as detailed in the interview scripts. If the probing does not result in an expanded answer, then a yes/no answer will suffice.  The interventions listed in the “what” section are based on a systematic review and meta-analysis completed by our research team. This systematic review evaluated the effectiveness of these interventions to rehabilitate people with cognitive deficits post-stroke. However, there remains unanswered questions about these interventions with regard to their optimal mode of delivery, timing of intervention etc. Where possible, these details will be gathered but we acknowledge that specifics of intervention design and delivery may not be possible to ascertain.

**Point 15:** Some of the questions are quite complicated. Would the participant be expected to know who would best deliver the intervention?

**Response 15: **Based on their experience of stroke rehabilitation as a person post-stroke, a caregiver or a health professional, their opinion on who they perceive as best to deliver the intervention would be valuable information for the design of a programme, where they have an opinion on this topic based on their experience. As discussed, we will pilot the interview on a participant from each stakeholder group and modify the script as required as well as allow for different administration between participants as their communication and cognitive issues require. Please see Methods, subheading: Piloting.

**Point 16:** How long on average are the interviews? Seems quite a substantial amount of material for someone with cognitive difficulty (and potential cognitive fatigue).

**Response 16: **Interviews are expected to last no more than one hour. Breaks will be given as required/requested, interviews may be conducted in 2-3 shorter sessions if required e.g. if cognitive fatigue is an issue. This has been addressed in the manuscript. Please see Methods, Data Collection, Line 317-319.

**Point 17:** Is the same guide used for focus groups? 19:

**Response 17: **Yes. This has been clarified in Methods. Please see Methods, Focus Groups, Line 357-358.

**Point 18:** *Guide for HCPs*- are you interested in “the ideal world” or what is feasible in their own clinical practice?

**Response 18: **We aim to explore both of these areas. As seen from our interview guide, we pose questions to gain perspectives on a hypothetical “ideal world” intervention. Given that there are substantial gaps in the literature to date regarding the optimal intervention to rehabilitate people with cognitive deficits post-stroke, we are interested in gaining these perspectives to fill the gap in the literature. In the closing questions of the interview, we pose a question regarding practicalities of delivering the intervention(s). Participants are encouraged to speak about their own clinical practice and what they perceive as working/ not working regarding the feasibility of interventions to rehabilitate cognitive impairment post-stroke. This will be vital to develop understanding of feasibility and acceptability of a potential intervention. Please see “interview guides” for further clarification on our line of questioning (available as extended data).

**Point 19:  **Data analysis:

 How will the separate data sets be integrated? Will the data be analysed separately for each of the groups or will this be integrated? Will interview and focus group data be analysed in the same way? Will member-checking only be used with proxy interviews? There is reference to "trustworthiness" but not adequate detail in relation to how this will be ensured. 

**Response 19: **Datasets from each stakeholder group will be analysed using reflexive thematic analysis. Therefore, each transcript will be analysed separately at first and then integrated in accordance with each stage of reflexive thematic analysis i.e. where data will be synthesised across groups. Yes, interview and focus group data will be analysed in the same way. We have added detail to the manuscript outlining how peer debriefing will be conducted to enhance the credibility and trustworthiness of data analysis. Please see Methods, Data Analysis, Line 437-439.

**Point 20:** How exactly will the findings be used to inform an intervention? This needs to be clearer given the title and aim of the research. The impact and need for the research should be clear

**Response 20: **Thank you. We have added a sentence to clarify the impact of these research findings. Please see Conclusion, Line 478-481.
